# Simultaneous beat-to-beat heart rate and systolic blood pressure variability in patients with and without neurally mediated syncope

**DOI:** 10.34172/jcvtr.2022.18

**Published:** 2022-06-21

**Authors:** Amirhossein Akbarzadeh, Fariborz Akbarzadeh, Babak Kazemi

**Affiliations:** ^1^Faculty of Medicine, Tabriz University of Medical Sciences, Tabriz, Iran; ^2^Cardiovascular Research Center, Tabriz University of Medical Sciences, Tabriz, Iran

**Keywords:** Syncope, Blood Pressure Variability, Tilt Test, Heart Rate Variability, Finapress

## Abstract

***Introduction:*** Autonomic changes play an essential role in the genesis of neurally mediated syncope (NMS). The aim of this study was to compare the changes of the autonomic nervous system (ANS) by measuring spectral indices of beat-to-beat systolic blood pressure and heart rate variability (SBPV and HRV) in ranges of low frequency (LF), high frequency (HF), and the LF/HF ratio during head-up tilt test (HUTT) in patients with and without a syncope response.

***Methods:*** In this case-control study of 46 patients with a suspected history of unexplained syncope, data were recorded separately during the typical three phases of HUTT. Patients who developed syncope were designated as the case group and the rest as the control group.

***Results:*** Thirty one patients experienced syncope during HUTT. Resting HRV and SBPV indices were significantly lower in cases than controls. After tilting in the syncope group, both HF and LF powers of SBPV showed a significant and gradual decrease. LF/HF in HRV increased in both groups similarly during the test but in SBPV, mainly driven by oscilations in its LF power, it increased significantly more during the first two phases of the test in syncope patients only to paradoxically decrease during active tilt (*P*< 0.001).

***Conclusion:*** Our findings show an abnormal autonomic function in patients with syncope, both at rest and tilting. Fluctuations of spectral indices of beat-to-beat SBPV, a potential noval index of pure sympathetic activity, show an exaggerated response during tilt and its withdrawal before syncope.

## Introduction

 Syncope is defined as a rapid and transient loss of consciousness due to a brief decline in cerebral blood flow with spontaneous and complete recovery after falling. One to two percent of emergency visits are due to syncope, and about half of these patients are admitted to the hospital.^1^ Neurally mediated syncope (NMS) accounts for 55-60% of all unexplained syncope cases.^2^ It has been believed that the main cause of NMS is the stimulation of the parasympathetic system due to the Bezold Jarish reflex in the empty left ventricle. Subsequently, severe decrement in blood pressure (BP) and or heart rate (HR) causes loss of consciousness and fainting. Although this mechanism is generally accepted, this theory was questioned by findings after cardiac transplantation.^3^ On the other hand, the neurohormonal mechanism may start the cascade of events that eventually cause syncope.^4^

 BP and HR constantly oscillate over time under the influence of control mechanisms such as autonomic nervous system (ANS), humoral factors, and respiration for maintaining cardiovascular homeostasis. Among the methods used for analyzing these oscillations, power spectural analysis of heart rate variability (HRV) has become an increasingly widespread guide for the assessment of ANS characteristics such as parasympathetic/sympathetic function or sympathovagal balace ^5,6^. The breath-dependent high frequency (HF) oscillations of HR give information about the parasympathetic (cardiovagal) activity and are usually about 0.2 – 0.3 Hz according to the usual breathing frequency (one breath every 3rd to 5th second). However, the low frequency (LF) oscillations of HR which has its summit at approximately 0.1 Hz are not a reliable index for the pure sympathetic activity, since different variables are involved in their origin. They become influenced by i.e. changes in the venous return, cardiac filling, the baroreceptor reflexes and the cardiac sympathetic activity.^7,8^ In contrast, information regarding ANS provided by SBPV is different from that provided by HRV. SBPV can also be analyzed on the basis of LH and HF of the power spectrum but it has been believed that the LF power of SBPV may be mediated primarily by the sympathetic nervous system.^9^ This sympathetic drive can be easily measured by the Task Force® Monitor software from the SBPV.

 Conflicting data have been reported on autonomic control during the HUTT phases prior to syncope, some reporting decreased sympathetic activity,^10^ some showing increased sympathetic activity,^11^ one describing both patterns^12^ and two studies reporting increased parasympathetic activity.^13,14^ A better understanding of the pathophysiology of tilt induced syncope could provide a more rational basis for the therapy of syncope.

 Searching literature showed few published articles with simultaneous evaluation of beat-to-beat HRV and SBPV in patients with NMS.^15-16,17^ To enrich the relevant data on mechanisms of NMS in adults, especially in the field of beat-to-beat SBPV, we decided to conduct this study. The primary goal of this study was to investigate the changes in HRV and SBPV during HUTT in patients with and without syncope. The results of this study can be used to help clarify the role of the ANS, specially the sympathetic nervous system by measuring the LF power of beat-to-beat SBPV, in the genesis of NMS. The availability of the Task Force® Monitor which performs simultaneous beat-to-beat HRV and SBPV analysis during phases of HUTT is an excellent opportunity in this regard.

## Materials and Methods

 In this case-control study between April 2018 and April 2019, patients with a suspected history of unexplained syncope during the previous six months, aged 18 to 40 years who accepted to participate in this study, were prospectively recruited. The LF and HF powers and their ratio of HRV and SBPV were recorded simultaneously during the typical three phases of HUTT (rest, passive tilt, and active tilt with nitroglycerine). Patients who developed syncope were designated as the case group and the rest as the control group. Subjects with diabetes, movement disorders such as Parkinson’s disease and multiple systemic atrophy, congestive heart failure of any cause, ischaemic cardiomyopathy and previous myocardial infarction, severe valvular heart disease, severe pulmonary hypertension, carotid sinus hypersensitivity, uncontrolled systemic hypertension, and severe chonic kidney disease were excluded because the HRV pattern could have been affected by their underlying disease. Drugs which interact with autonomic modulation were discontinued for at least five half-lives before the day of testing. Patients with a history of psychiatric illness who were on psychiatric drugs or had permanent atrial fibrillation, grade two or three atrioventricular-nodal block, complete bundle branch block, pacemaker rhythm, frequent extra-stimulus in resting ECG, or signs of channelopathies in resting ECG, and those who were reluctant to complete the test were also excluded from the study. After taking a complete history and physical exam, data on baseline characteristics of patients including age, sex, height, weight, and full drug history were collected. ECG and echocardiographic study were done for all patients. Informed consent for doing the test and attending the research protocol was obtained from all patients. HUTT was performed according to the Italian protocol^18^ after at least four hour fasting in the afternoon in a quiet room with dimmed lights and adjusted temperature around 24°C. A skilled nurse prepared the patients, and the tests were done under the direct supervision of a cardiologist. The test was performed by means of an electrically controlled tilt table with a footboard for weight bearing. After a resting period of 6 minutes in the supine position, the patient was tilted to 70° for 25 minutes (passive phase). Thereafter, in the absence of spontaneous syncope, 400 µg of nitroglycerine spray were administered subligually and the patient remained tilted for an additional 20 minutes (provocation phase), unless syncope occurred. The test was considered completed either with the occurrence of syncope or ending of the provocation phase in the absence of symptoms. The haemodynamic response to HUTT was classified according to the VASIS classification [cardioinhibitory (CI), vasodepressor (VD), or mixed types].^19^

 Surface ECG and beat-to-beat SBPV and HRV were simultaneously recorded using the Task Force® Monitor System (CNSystems Medizintechnik GmbH, Austria). The Task Force® Monitor System is a device for continuous noninvasive assessment of hemodynamic and autonomic regulation of the cardiovascular system and is particularly useful in the diagnostic processing of patients with syncope. With the special software, ECG, HR, and BP are recorded and visualized during the HUTT and then all the data are exported for evaluation. BP was measured beat-to-beat on the finger artery with a suitable sized double finger sensor attached to the third and fourth fingers of the right hand, using CNAP technology. With the so-called vascular unloading technique, plethysmographic signals are transformed into continuous BP information. The so-called VERIFI-algorithm is applied to overcome the problem of bias caused by vasoconstriction or vasodilatation.^19^ A separate left-arm pressure cuff was applied to take BP every 5 minutes. Recordings with artefacts in a signal involved in total more than 10% of values were excluded from further analysis; signals with less than 10% artefacts were edited (the erroneous values were replaced by the median of the surrounding seven correct values).^20^ Frequency power indices of both HRV and SBPV, including LF, HF, and LF/HF ratios, were recorded. The original recorded files were in the format of text files which were converted to data files for further analysis. In the first step of the analysis, the entire beat-to-beat data were analyzed and summarized for all study groups with and without syncope. In the second stage of the study, beat-to-beat data were divided into 30 seconds blocks and the mean of each block was used for drawing more informative figures. Finally, we compared the HRV and SBPV indices separately between case and control groups.

###  Statistical analysis

 The Excel software for windows was used for data collection and entry. Continuous quantitative data were expressed as mean ± SD and compared between groups by student t-test and the ANOVA test. A comparison of quantitative data in each group at different phases of HUTT was made by paired samples t-test. Qualitative data were presented as frequency, and no comparisons were made. A P-value of less than or equal to 0.05 was considered a statistically significant difference between variables. Figures were drawn by Powerpoint 2016 software for windows. We used two statistical softwares, SPSS 22 for windows and web-based open EPI Info in this study.

## Results

###  Baseline characteristics and general information

 Fifty three of seventy two patients who were referred to our outpatient syncope clinic between April 2018 to April 2019 were eligible to participate in this study. Three patients withdrew their consent to take part in the study and the data on four patients were corrupted. Finally, 46 patients (31 patients in case and 15 patients in the control group) were analysed. Baseline characteristics of patients are summarised in Table 1. Because of the strict inclusion and exclusion criteria, all patients had a healthy heart. Therefore echocardiographic and electrocardiographic results of patients are not shown.

**Table 1:** Baseline characteristics of patients

**Groups (no)**	**Male/female**	**Mean Age Y (SD)**	**Mean Height CM (SD)**	**Mean Wight KG (SD)**	**Body surface area m^2^ (SD)**
No Syncope (15)	13/2	27.13 (6.46)	173.80 (9.45)	68.73 (15.02)	1.82 (0.21)
Syncope (31)	16/15	29.16 (8.97)	170.55 (9.68)	75.23 (13.48)	1.82 (0.20)
Cardioinhibitor Syncope (12)	8/4	28.33 (7.94)	172.42 (10.05)	8.67 (15.61)	1.93 (0.21)
Mixed Type Syncope (12)	4/8	30.67 (10.12)	168.00 (9.04)	74.00 (8.50)	1.80 (0.17)
Vasodepressor Syncope (7)	4/3	28.00 (9.59)	171.71 (10.59)	68.00 (12.39)	1.79 (0.19)
Significance (Syncope/No Syncope)	0.02	0.43	0.28	0.14	0.66

 In the case group, 9 (29.1%) developped syncope in the passive phase of the test ( 6 CI, 1 VD, and 2 mixed type). Twenty two patients (70.9%) developped syncope in the active pase of the test (6 CI, 6 VD, and 10 mixed type).

###  Fluctuations of HRV during HUTT

***Phase 1***: Patients in the syncope group showed a significantly lower LF and HF flactuations and higher LF/HF ratio with respect to the controls (*P* < 0.001).

***Phase 2***: In comparison with the resting state, LF activity did not change but HF activity decreased and the LF/HF ratio increased significantly in both groups (*P* < 0.001). During tilt, LF activity was not statistically different between groups (*P* = 0.8) but HF activity was significantly lower in cases (*P* = 0.04). The LF/HF ratio was not different between study groups (*P* = 0.9)

***Phase 3:*** There was no statistical difference in LF (*P* = 0.8), HF (*P* = 0.3), and LF/HF (*P* = 0.2) ratio between groups during phase 3 of HUTT (Table 2).

**Table 2:** Comparison of mean (SD) HRV (ms^2^) and SBPV (mmHg^2^) indices in patients with and without syncope

**Index/Group**		**HRV**					**SBRV**				
		**control** **15**	**CI** **12**	**VD** **7**	**Mixed** **12 **	**Case 31**	**control** **15**	**CI** **12**	**VD** **7**	**Mixed** **12 **	**Case ** **31**
LF	Rest	461 (69)	294 (71)*	277 (50) *	500 (290)	371 (113) ¶	14.28 (0.93)	8.16 (0.77)*	6.13 (0.23)*	4.71 (0.52)*	6.38 (0.45) ¶
	Tilt 70	529 (1936)	593 (9569)	239 (1166)	246 (583)*	363 (3330)	10.39 (1.22) **	7.08 (0.74)*	6.26 (0.48)*	4.39 (0.39)*	5.81 (0.39) ¶ **
	TNG	535 (434)	611 (740)	27.44 (56.11) *	338 (610)	435 (2011)	7.33 (0.76)	3.92 (1.1)*	1.93 (1.1)*	3.84 (0.52)*	3.52 (0.47) ¶
HF	Rest	675 (52)	189 (23)*	334 (49)*	604 (389)	384 (136) ¶	4.17 (0.09)	1.96 (0.13)*	1.19 (0.08)*	1.03 (0.04)*	1.43 (0.06) ¶
	Tilt 70	118 (80) **	75 (51)	51 (30) *	91 (110)	75 (61) ¶ **	2.33 (0.29) **	1.16 (0.7)*	1.11 (0.14)*	0.74 (0.7)*	0.99 (0.04) ¶ **
	TNG	84 (26)	155 (218)	5.42 (7.03) *	203 (467)	134 (198)	1.29 (0.18)	0.97 (0.22)*	0.48 (0.16)*	0.69 (0.15)*	0.73 (0.13) ¶
LF/HF	Rest	0.68 (0.12)	1.58 (0.45)*	0.83 (0.13)*	1.00 (0.32)*	0.99 (0.13) ¶	3.42 (0.23)	4.14 (0.14)*	5.16 (0.36)*	4.57 (0.46)*	4.43 (0.15) ¶
	Tilt 70	4.29 (1.52) **	5.10 (2.03)	4.30 (3.17)	3.76 (3.41)	4.34 (1.67) **	4.48 (0.47) **	6.09 (0.80)*	5.62 (0.95)*	5.88 (0.92)*	5.86 (0.49) ¶ **
	TNG	5.42 (2.6)	5.13 (2.05)	2.99 (3.60)	4.44 (2.59)	4.48 (2.15)	5.80 (0.76)	4.08 (0.99)*	4.02 (1.58)*	5.57 (1.44)*	4.84 (0.65) ¶

Abbreviations: HRV, Heart rate variability; SBPV, Systolic blood pressure variability; CI, Cardioinhibitory; VD, Vasodepressor; Mixed, Mixed type; LF, Low frequency in HRV by ms^2^ and in SBPV by mmHg^2^; HF, High frequency in HRV by ms^2^ and in SBPV by mmHg^2^ *Significant difference between control and each syncope groups **Significant difference resting and tilt 70 values ¶Significant difference between case and control group

###  Fluctuations of SBPV during HUTT

 As shown in Table 2;

***Phase 1***: LH and HF powers of SBPV had lower flactuations and the LF/HF ratio was higher in cases vs. controls (*P* < 0.001).

***Phase 2***: The fluctuations of LF, HF, and LF/HF ratio was similar to phase 1.

***Phase 3***: LH and HF powers of SBPV had lower flactuations and the LF/HF ratio was lower in cases vs. controls (*P*< 0.001).

**Figure 1:**
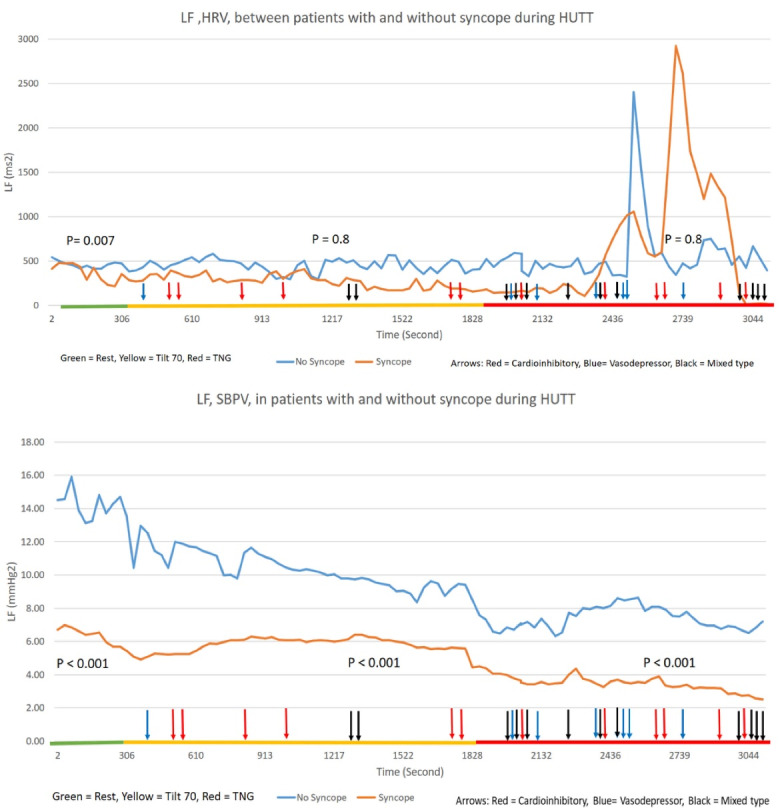
Comparison of LF in HRV and SBPV in patients with and without syncope during HUTT. Arrows: Time of syncope, LF= Low frequency, HRV= Heart rate variability, SBPV= Systolic blood pressure variability, HUTT= Head up tilt test.

**Figure 2:**
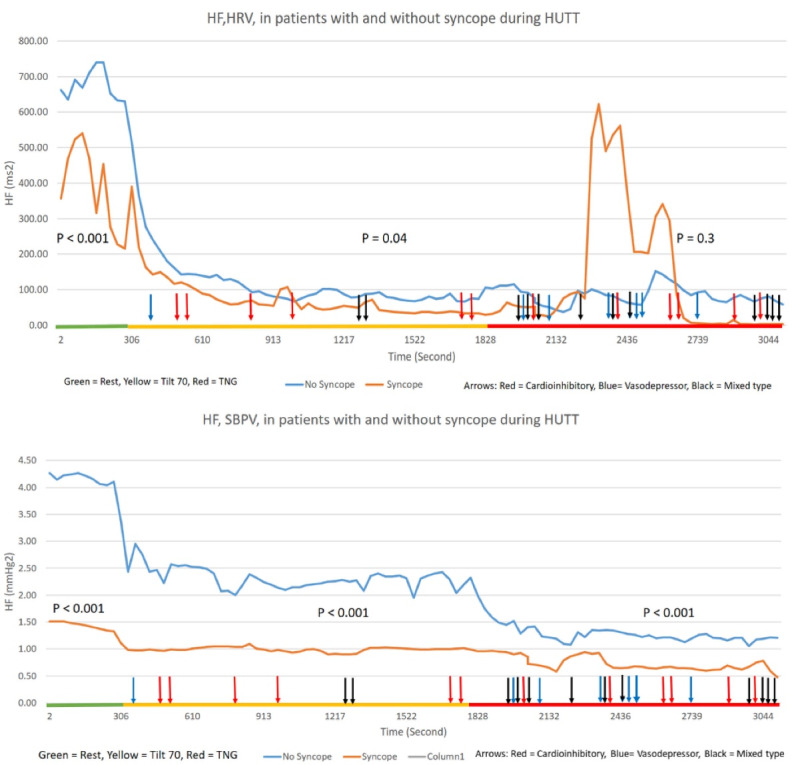
Comparison of HF in HRV and SBPV in patients with and without syncope during HUTT. Arrows: Time of syncope, HF= High frequency, HRV= Heart rate variability, SBPV= Systolic blood pressure variability, HUTT= Head up tilt test.

**Figure 3:**
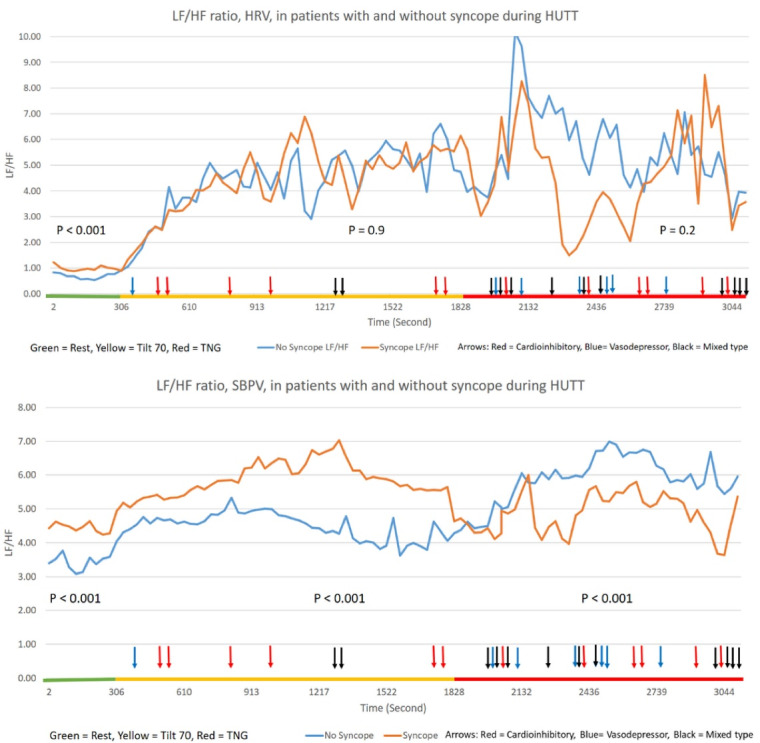
Comparison of LF/HF ratio in HRV and SBPV in patients with and without syncope during HUTT. Arrows: Time of syncope, LF= Low frequency, HF= High frequency, HRV= Heart rate variability, SBPV= Systolic blood pressure variability, HUTT= Head up tilt test.

 For a better understanding of results, the entire data were divided into consecutive 30 seconds blocks during each phase of HUTT, the mean values of each block were calculated, and separate graphs for LF, HF, and LF/HF ratio fluctuations in HRV and SBPV in cases and controls were drawn. (Figures 1-3). Figure 1 upper and lower panel shows the fluctuations of LF between case and control groups in HRV and SBPV, respectively. In addition to the findings mentioned above, the dominant result is the slow fluctuations of LF in HRV during phase 2. In phase 3, fluctuations increased in both groups without significant difference. Conversely, in SBPV, there was a continuous decrement in LF power, which was shown in both groups in all phases of HUTT. Figure 2 upper and lower panel shows the fluctuations of HF between case and control groups in HRV and SBPV, respectively. After tilting, the activity of HF decreased significantly in HRV and SBPV. After that, HF activity remained low in both groups; the exception was increased activity during the early minutes of phase 3 in the HRV power of syncope patients. Figure 3 upper and lower panel shows the fluctuations of the LF/ HF ratio between cases and controls in HRV and SBPV, respectively. During phase 1 and 2 of the test, the LF/HF ratio of SBPV, although lower in controls compared to cases, increased in both groups similarly during the test, but just after starting phase 3, there was a rapid reversal of this ratio in syncope patients mainly driven by oscilations in its LF power.

- The upper panel shows the LF (ms^2^) activity among case and control groups in the field of HRV, which shows the low activity of LF at the beginning of the HUTT in the case group (P < 0.001). After that, while the activity remains unchanged in the control group, in the case group, the activity became smaller gradually during the entire phases of 1, 2, and up to mid-time phase 3 (the difference between groups was not significant). In the second half of phase 3, in 11 patients who developed syncope, the activity of LH increased significantly, even more than the control group. During the last seconds of HUTT, the LH activity in the case group fell to a very low and undetectable level.
- The lower panel shows the LF (mmHg^2^), SBPV, activity among case and control groups. LF was lower significantly in the case than the control group. With advancing the HUTT, the activity of LF decreased in both groups. This decrement was smaller in the case group during phase 2 of the study. The differences between study groups were significant in all phases of the study (P < 0.001).

- The upper panel compares HF (ms^2^) of HRV. During the initial resting phase of the study, the HF was lower in the case than the control group. Soon after starting phase 2 of HUTT (passive tilt 70°), HF activity decreased dramatically in both case and control groups. The level of activity was low in both groups during phase 2 of the study. After provocation with TNG, in the case group, the HF activity jumped to a very high level transiently, but after about 10 minutes fell to an undetectable level. The differences in HF activity were significant in phase 1 and 2 between the case and control group P < 0.001.
- The lower panel shows HF (mmHg^2^) in SBPV among case and control groups. Like HF in HRV, HF activity in SBPV was lower in the case than the control group, P < 0.001. Starting phase 2 of the study was accompanied by the rapid falling of the activity of HF in both groups, which was more prominent in the control group. The activity of HF in both groups showed a plateau during phase 2 of HUTT. Again the difference between the two groups was significant P < 0.001. HF decreased rapidly with the starting of phase 3 of HUTT. This decrement was higher in the control group. During the rest of this phase, the case and control groups showed a plateau activity of HF. The difference was significant between groups, P < 0.001.

- The upper panel of this figure shows the LF/HF ratio of HRV in case and control groups. Unlike the LF and HF, in the resting phase of HUTT, LF/HF ratio was lower in the control group, P < 0.001. With the beginning of phase 2, the LF/HF ratio increased gradually and reached its plateau after about 8 minutes. The difference between the case and control groups was not significant. Phase 3 was accompanied by fluctuations in the LF/HF ratio in both groups that were not significant.
- The lower panel shows the LF/HF ratio in the field of SBPV. The LF/HF ratio was significantly lower in the control group than the case group in the first phase of the study (P < 0.001). The first half of Phase 2 of HUTT displays a slight increase in the LF/LH ratio in both study groups. The increment was higher in the case group. During the second half of this phase, the LF/HF ratio decreased gradually in both groups. The difference was significant in this phase (P < 0.001). The beginning of phase 3 was accompanied by further lowering of the LF/HF ratio in the case group, while the ratio increased in the control group. In the first half of this phase, the direction on the LF/HF ratio was discordant in study groups. The second half of this phase showed plateau activity of the LF/HF ratio in both groups. The difference was significant between study groups (P < 0.001).

## Discussion

 This study is one of the few studies that evaluated simultaneous beat-to-beat HRV and SBPV in patients with and without syncope during the HUTT. The main result of this study, aside from showing the feasibility of these mesurements dring HUTT, was the clarification of LF fluctuations of SBPV during syncope. As mentioned before, the LF power of the HRV is under the influence of many factors like venous return, cardiac filling, the baroreceptor reflexes, and the cardiac sympathetic activity.^8,21^ However, the LF power in SBPV is the pure reflection of the sympathetic nervous system activity ^9^. We showed (Figure 1) that LF fluctuations in SBPV continuously decreased in all stages of HUTT in both case and control groups but with a steeper decrease in patients with syncope. This finding can help to improve our knowledge about the exact role of sympathetic activity during syncope and the phenomenon of sympathetic withdrawal.^21^

***Phase 1 (Supine):*** patients included in this study were otherwise healthy people without any comorbidities. Considering the normal values of HRV indices reported by Nunan et al^22^ the LF and HF powers of our patients in the control group were within the normal limit. Conversely, patients with syncope had lower than normal values that were compatible with the findings of other studies.^23,24^ In SBPV analysis, the differences in flacuations of LF and HF in each group of patients were discordant and statistically significant. On the other hand, the fluctuations in LF and HF powers of HRV were concordant and statistically similar (Figures 1,2). The reason for this phenomenon needs to be elucidated further but clearly it hints different mechanisms for producing these flactuations in HRV vs. SBPV.

***Phase 2 (Passive Tilt):*** the LF power of SBPV showed continuous decrement during this phase, which was different from the plateau pattern of LF power of HRV. Conflicting results about the LF power activity during the passive phase of HUTT has been reported, showing plateau responses,^25^ increased activity,^26^ and decreased activity.^27^ Therefore the pattern of LF power activity in SBPV may be a correct response because it is not under the influence of other factors like baroreceptor sensitivity. Moke et al reported the same results in their study on children with syncope.^3^ TheHF power, which is a marker of parasympathetic activity in HRV and SBPV,^28,29^ decreased significantly in this phase in acordance by the findings of other studies.^30^ Lowering of parasympathetic activity may act as a protective mechanism against developing syncope. Previous studies have sugested the essential role of increased parasympathetic activity in the genesis of syncope.^31^ Nine of our patients developed syncope during this phase while the HF power was very low chalenging the original role of parasympatetic activity in syncope. While the LF/HF ratio increased significantly in both HRV and SBPV, the difference between case and control was not significant statistically in HRV, and curves had an overlap on each other (Figure 3). Efremov et al reported in their study that the LF/HF ratio is not different between case and control groups.^26^ Therefore when the LF flactuations in HRV are not a reliable marker of sympathetic activity, it is better to use LF/HF ratio in SBPV as a marker of sympathovagal balance in which the LF component is a pure reflection of sympathetic activity. In this regard, decrement of the LF/HF ratio in the second part of HUTT in the case group can be a sign of autonomic insufficiency.

***Phase 3 (Active Tilt):*** a small and non-significant increase in LF power of HRV and SBPV was seen, that may be the effects of nitroglycerine, which decreases baroreceptor sensitivity and increases sympathetic activity.^32^ Non-significant changes of HF power activity (both HRV and SBPV) is aligned with the findings that sublingual nitroglycerine makes little changes in parasympathetic activity and tends to decrease it.^32^ Twenty-two of our patients developed syncope in this phase. Sixteen of theses syncopes were vasodilatory or mixed type, which share the component of vasodilatation. Sublingual nitroglycerine provokes syncope by decreasing the stroke volume and inducing vasodilatation.^33^ Therefore these subtle changes in HRV and SBPV indices most probably are not related to the effects of nitroglycerine on the ANS but on the other hand might be linked to the failure of the ANS to tolerate to the aditional hemodynamic burden imposed by nitroglycerine in patients with syncope.

 This study may suffer from some limitations:

1. Patients in the control group were not normal people without a history of syncope. The respective positivity rate with HUTT in subjects without syncope ranges from 11 to 14%.^34^ On the other hand patients with history of unexplained syncope and negative HUTT usually have bradycardia or transient complete heart block and the mechanism of syncope is not neurally mediated.^35^ Performing HUTT in patients without syncope is ethically unacceptable. Patients in the control group had normal ranged HRV indices, and there were continuous and significant differences between HRV indices between the case and control groups at rest. Therefor we assume that the effects of our control in the results of the study were negligible.
2. The sample volume was too small to analyse the results in patients with different types of syncope.
3. The time of performing HUTT in our study was in the afternoon. There is a diurnal variation in the occurrence of syncope, more prevalent during the morning.^36^ Therefore we may face some limitations in this regard.
4. Because of the low sample volume, we didn’t analyse the results based on gender.

## Conclusion

 Our findings show an abnormal autonomic function in patients with syncope, both at rest and tilting. Fluctuations of spectral indices of beat-to-beat SBPV, a potential noval index of pure sympathetic activity, show an exaggerated response during tilt and its withdrawal before syncope. Real-time spectral analysis of HRV and SBPV is feasible during HUTT, and the results of each method may open new insights into the mechanisms behind NMS.

## Acknowledgments

 We want to give the best of our thanks to the patients who participated in this study and to Tabriz University of Medical Sciences for approving it. Also, we appreciate our electrophysiology nurse, Maryam Seidgar, for her help in this study,

## Funding

 Cardiovascular research center of Tabriz University of Medical Sciences funded this study.

## Ethical approval

 The ethical committee of Tabriz University of Medical Sciences approved this study (approval ID: IR.TBZMED.REC.1397.602).

## Competing intrest

 The authors of this article reported no any competing intrest.
